# Panicle transcriptome of high-yield mutant *indica* rice reveals physiological mechanisms and novel candidate regulatory genes for yield under reproductive stage drought stress

**DOI:** 10.1186/s12870-023-04507-1

**Published:** 2023-10-13

**Authors:** Aparna Eragam, Ankita Mohapatra, Vishnu Shukla, Rajashekar Varma Kadumuri, Abin Panackal George, Latha Putta, Srividhya Akkareddy, Sreenivas Chavali, Lakshminarayana R. Vemireddy, Eswarayya Ramireddy

**Affiliations:** 1 Department of Biology, Indian Institute of Science Education and Research (IISER) Tirupati, Tirupati, 517507 Andhra Pradesh India; 2 Department of Molecular Biology and Biotechnology, S.V. Agricultural College, Acharya NG Ranga Agricultural University (ANGRAU), Tirupati, 517502 India; 3 Regional Agricultural Research Station (RARS), ANGRAU, Tirupati, India

**Keywords:** Gamma-irradiated mutant, *Oryza sativa* L. ssp. *indica*, Panicle development, Reproductive stage drought stress, Rice, Yield under drought

## Abstract

**Background:**

Reproductive stage drought stress (RDS) is a major global threat to rice production. Due to climate change, water scarcity is becoming an increasingly common phenomenon in major rice-growing areas worldwide. Understanding RDS mechanisms will allow candidate gene identification to generate novel rice genotypes tolerant to RDS.

**Results:**

To generate novel rice genotypes that can sustain yield under RDS, we performed gamma-irradiation mediated mutation breeding in the drought stress susceptible mega rice variety, MTU1010. One of the mutant *MM11* (MTU1010 derived mutant11) shows consistently increased performance in yield-related traits under field conditions consecutively for four generations. In addition, compared to MTU1010, the yield of *MM11* is sustained in prolonged drought imposed during the reproductive stage under field and in pot culture conditions. A comparative emerged panicle transcriptome analysis of the MTU1010 and *MM11* suggested metabolic adjustment, enhanced photosynthetic ability, and hormone interplay in regulating yield under drought responses during emerged panicle development. Regulatory network analysis revealed few putative significant transcription factor (TF)-target interactions involved in integrated signalling between panicle development, yield and drought stress.

**Conclusions:**

A gamma-irradiate rice mutant *MM11* was identified by mutation breeding, and it showed higher potential to sustain yield under reproductive stage drought stress in field and pot culture conditions. Further, a comparative panicle transcriptome revealed significant biological processes and molecular regulators involved in emerged panicle development, yield and drought stress integration. The study extends our understanding of the physiological mechanisms and candidate genes involved in sustaining yield under drought stress.

**Supplementary Information:**

The online version contains supplementary material available at 10.1186/s12870-023-04507-1.

## Background

Rice is one of the most important staple cereal crops globally [1]. With a steadily increasing world population, rice consumption is projected to increase by approximately 100 million tons by 2050 (Food and Agriculture Organization of the United Nations, 2020). Enhancing rice production is an immediate challenge to sustain food balance in the coming future. Rice is well adapted to waterlogged conditions, due to which sensitivity to soil water content is very high [2, 3]. Rice production is severely affected by drought stress in most of the cultivation fields around the world, primarily due to increasing water scarcity [4]. Although rice is affected by drought at all phenological growth stages, the impact of drought especially at reproductive growth affecting panicle and grain development is more threatening as it could lead to yield losses upto 60% [5–7]. Given this magnitude of impact, it is paramount to understand reproductive stage drought response mechanisms and the development of drought-tolerant and water use-efficient rice varieties.

Depending on the developmental growth stage, drought stress triggers various morpho-physiological responses. Generally, in most cereals, drought limits the photosynthetic performance, signal transduction (mainly, osmotic and hormonal) and carbohydrate metabolism, thereby reducing the yield [8–10]. Several quantitative trait loci (QTLs) for rice vegetative drought tolerance have been identified. These are governed by contribution from many loci regulating drought-responsive growth parameters that integrate photosynthesis, Abscisic acid (ABA) and water relations. Several gene expression and transgenic-based studies have identified important drought-tolerant genes. For example, late embryogenesis abundant (*LEA*) proteins [11], ascorbate peroxidase (*APX*) [12], AP2/ERF family members (*OsERF48* and *OsERF71*) [13, 14], bZIP family members (*OsbZIP12* and *OsbZIP71*) [15, 16], MYB family members (*OsMYB2* and *OsMYB6*) [17, 18] and NAC family members (*OsNAC5*, *OsNAC6* and *OsNAC14*) [19, 20] have been shown to be involved in rice drought tolerance. Various spatio-temporal based transcriptomic studies have identified differentially expressed drought-responsive genes between contrasting rice varieties to drought [21–25]. These studies highlighted the role of a few transcription factors (TFs) that are involved mainly in processes affecting osmoregulation and reactive oxygen species (ROS) scavenging during vegetative drought stress. However, only a few genes have been implicated in reproductive drought stress tolerance in rice. For instance, a drought-inducible AP2/ERF transcription factor (TF), *OsAP37* was able to recover rice from dehydration during vegetative growth and reduced the overall yield loss at the reproductive stage [26]. Overexpression of NAC-TF family member *OsNAC19* significantly enhanced the seed setting by rapid stomatal closure and turgor pressure management [27]. ROS-scavenging capability also sustained drought response during reproduction when Ski-interacting protein (*OsSKIPa*) was constitutively expressed in rice [28]. Moreover, drought stress during rice panicle development dramatically affects male sterility by affecting hormone-regulated programmed cell death (PCD) in anthers [29]. Both ABA and gibberellic acid (GA) have been shown to affect flower development during reproductive drought stress [30].

Though the aforementioned studies shed light on biochemical and hormonal players involved in drought response at vegetative stage, there is a huge void regarding our understanding about the transcriptional regulation of rice yield, resulting from the effects of drought stress at reproductive stages. The continuous artificial selection and breeding of rice during domestication processes has led to the generation of varieties with varying levels of yield and drought susceptibility [31]. MTU1010 or Cotton Dora Sannalu is one of the widely cultivated mega rice varieties known for its high yield, short duration, alluring long slender grain type [32]. MTU1010 is highly sensitive to drought, which leads to significant yield losses every year [33, 34]. In the present study, we performed mutational breeding in MTU1010 to identify the mutants with increased yield under well-watered (control) and drought conditions. In M4 and M5 generations, we identified a few mutant lines exhibiting better yield-related traits under control and reproductive drought stress. For a more comprehensive understanding of the drought-responsive mechanism during panicle development, a comparative panicle transcriptomic study was undertaken using MTU1010 and a mutated gamma-ray line (*MM11*) with a sustained yield under drought conditions. Furthermore, we performed panicle transcriptomics under drought stress and well-watered (WW) conditions and identified a set of yield (Y), drought (D) and Yield under drought (YD) differentially expressed genes (DEGs). Subsequent analysis reveals significant enrichment of distinct biological processes along with transcription factor (TF)-target interactions. The present study identifies a mutant line that sustains yield under drought conditions and provides novel candidates for improving MTU1010 against reproductive drought stress. It further enhances our understanding of drought tolerance and emerged panicle development in rice.

## Results

### Selection of high-yielding mutant lines

**Fig. 1:**
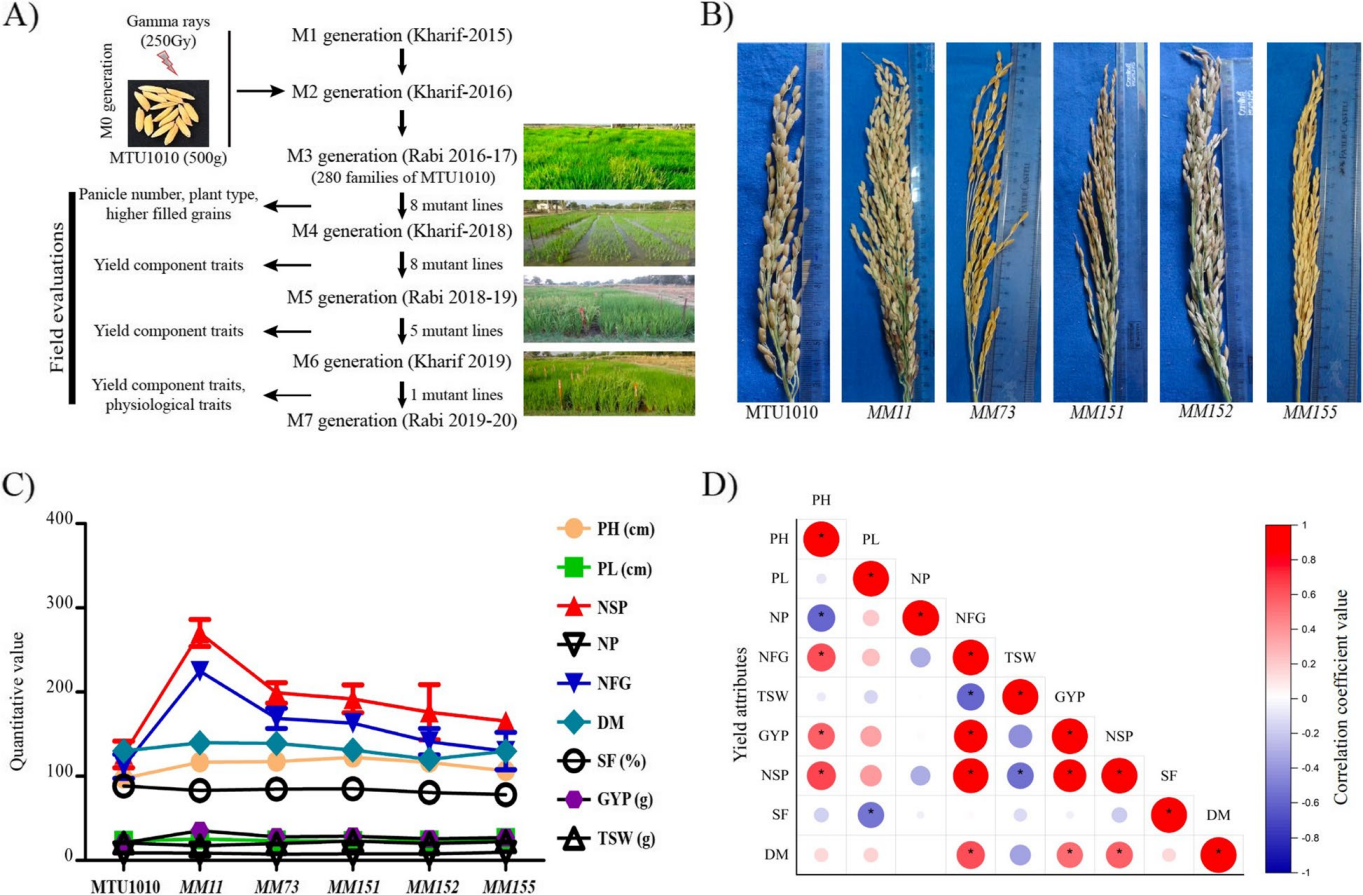
Yield-based phenotypic comparison of MTU1010 with mutant genotypes. **A** Schematic representation of the workflow for yield-based mutant characterization and drought tolerance evaluations. **B** Panicle phenotypes of MTU1010 along with five mutant genotypes. **C** Comparative plot of yield attributes from various seasons in MTU1010 and mutant genotypes (M5 generation). The X-axis and Y-axis represent genotypes and quantitative values, respectively. The data represented are means of three biological replicates. Vertical bars indicate the standard error. The complete statistical data is provided in Supplementary Table S1A. **D** Pearson correlation coefficients among different yield attributes (PH, plant height; PL, panicle length; NSP, number of spikelet/plant; NP, number of panicles; NFG, number of filled grains per panicle; DM, days to maturity; SF, spikelet fertility; TSW, thousand seed weight; GYP, grain yield/plant). * and circle size indicates level of significant correlation. *p ≤ 0.05

Gamma-irradiation induced genetic variation in plants has been shown to promote rice yield enhancement [35]. In this study, the M0, M1 and M2 generations were evaluated for various traits under normal irrigated condition. During the 2016–17 winter (rabi) season, yield-related traits of 280 M3 families were evaluated. Eight mutant lines were selected from 280 families based on their panicle architecture and number of filled grains. These eight mutant lines were further assessed for high yield under normal irrigated conditions during the (M4 generation) monsoon of 2018 (Kharif). On the basis of the consistency of yield-related traits, five mutant lines, *MM11*, *MM73*, *MM151*, *MM152*, and *MM155*, were selected and tested in M5 and M6 generations during winter 2018–19 and monsoon 2019 (Fig. 1A, B, and Table S1A). All five mutant lines showed a higher number of filled grains (NFG) and a higher number of spikelets per panicle (NSP) in comparison to MTU1010 (Fig. 1C). Pearson correlation of all the measured yield traits showed a significant positive correlation of NFG (*r* = 0.79, *p* < 0.05) and NSP (*r* = 0.73, *p* < 0.05) trait with grain yield per plant (GYP) (Fig. 1D). However, among the entire mutant lines tested, *MM11* plants showed stable superior performance across M4 (50.87%), M5 (29.12%), and M6 (69.91%) generations for grain yield per plant (GYP) trait (Table S1A). We selected *MM11* mutant for further studies because of its consistent yield performance under control conditions. The yield advantage of *MM11* is further tested by imposing the reproductive stage drought stress under field conditions for 21 days during winter (2018–19). The quantification of yield and yield-related traits under the field drought condition revealed that drought stress reduced NFG, TSW, SF and RWC for both MTU1010 and *MM11* (Table S1B). However, *MM11* maintained a higher grain yield (12.50%) under stress by keeping a high filled grain number per panicle (50%) than MTU1010 (Table S1B). Next, we investigated the physiological, biochemical and molecular response to reproductive drought stress in *MM11* genotype.

### *MM11* mutant sustained yield under reproductive drought stress

**Fig. 2:**
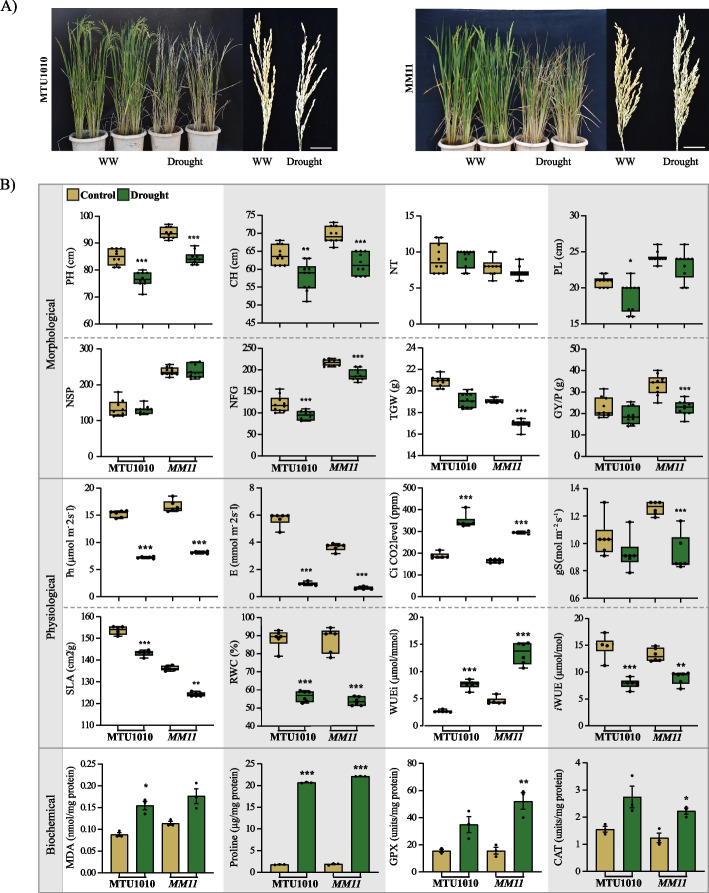
Physiological characterization of mutant (*MM11*) under reproductive stage drought stress (RDS) conditions. *MM11* plants (M7 Generation) showed better yield and physiological growth performance under stress conditions in comparison with MTU1010. **A** Phenotypic differences in MTU1010 and *MM11* plants under well-watered (WW) and moisture stress at reproductive stage and in mature panicles. **B** Quantitative differences in yield (PH, plant height; CH, culm height; NT, number of tillers; PL, panicle length; NSP, number of spikelet/panicle; NFG, number of filled grain/plant; TGW, thousand grain weight; GYP, grain yield/plant); photosynthesis (Pn, photosynthesis rate; Ci, intercellular CO_2_ level; gs, stomatal conductance; SLA, specific leaf area; RWC, relative water content; WUEi, intrinsic water use efficiency; iWUE Instantaneous water use efficiency) and anti-oxidant (MDA, malondialdehyde; Proline; GPX, guaiacol-peroxidase; CAT, catalase attributes during *MM11* and MTU1010 growth under RDS. *indicates significant variation in drought when compared to well-watered (WW) values. Data represented in the figure are means of six to twelve biological replicates. Vertical bars indicate the standard error. The complete statistical data is provided in Supplementary Table S1C. *p ≤ 0.05; **p ≤ 0.01; ***p ≤ 0.001

After field evaluations, the *MM11* mutant plants were further characterized and compared to MTU1010 for yield-related traits, photosynthesis performances and antioxidant activity during the reproductive stage drought stress under pot culture grown conditions. Consist with the previous generation's performance at the field level, the *MM11* genotype showed enhanced performance in yield-related traits such as plant height (PH), panicle length (PL), number of filled grains per panicle (NFG), number of spikelets per panicle (NSP), spikelet fertility (SF) and grain yield per plant (GYP) compared to its parent MTU1010 under the well-watered (WW) condition in pot culture grown conditions (Figs. 2A, B and Table S1C). For example, under well-watered conditions, consistent with field growth, an increment (47.38%) of GYP in *MM11* (33.50 g) over MTU1010 (22.73 g) was observed. In addition, *MM11* showed high NFG and NSP in both control and stress conditions than MTU1010. However, drought stress at the reproductive stage did not significantly affect the number of tillers (NT) and panicle length (PL) in *MM11*. Overall, under stress condition, improved yield levels in *MM11* compared to MTU1010 was found to be supported by a moderate gain in PH (10.29%), spikelet fertility (8.1%) and a higher level of gain in PL (22.87%), NFG (99%), NSP (85%), along with the final GYP (38.29%) (Fig. 2B and Table S1C).

During drought stress, water status, photosynthesis, gaseous exchanges and assimilate partitioning are major physiological processes that are severely affected and responsible for the reduction in yield. As expected, prolonged drought stress causes reduced relative water content (RWC) in both *MM11* (38.93%) and MTU1010 (36.12%) leaves to their respective well-watered condition, which directly reflects the drought effect on plants (Fig. 2B). Similarly, a reduction of specific leaf area (SLA) was also observed in *MM11* (13.12%), and MTU1010 (11.29%) stressed plants indicating the severity of the drought on the genotypes tested (Fig. 2B). Furthermore, we analysed the effect of reproductive stage drought stress (RDS) on photosynthetic parameters. The results showed that RDS causes a reduction in the photosynthetic rate (Pn) of *MM11* and WT (MTU1010) (Fig. 2B). However, *MM11* showed about 3% higher in Pn under both well-watered and stress conditions compared to parent MTU1010. The rise in Pn of *MM11* might be due to its increased stomatal conductance (gs) under well-watered and drought conditions. This high gs under drought led to a marginal increase in intercellular CO_2_ levels (Ci) in *MM11* (44%) compared to MTU1010 (84%) under drought stress conditions. A significant increase in the instantaneous water use efficiency (WUEi), was observed in *MM11* compared to MTU1010 under both well-watered (WW) (40%) and stressed (48%) conditions (Fig. 2B), majorly due to sustained photosynthetic activity with respect to relatively low transpiration rate. However, intrinsic water use efficiency (iWUE) was not affected due to unaltered stomatal conductance. This result is consistent with previous studies, where drought-tolerant genotypes possess an increased WUEi than drought-susceptible soybean genotypes [36]. Next, the levels of proline and malondialdehyde (MDA) were quantified in response to RDS. It has been shown that an increase or decrease in proline and MDA levels correlate to rice genotypes' drought tolerance [37]. In response to drought, both genotypes respond similarly in terms of Proline and MDA content by showing a significant increase compared to their respective well-watered (WW) conditions (Fig. 2B). This suggests that the sustained yield of *MM11* is not due to the better maintenance of membrane integrity or osmatic balance. Further, we quantified the effect of RDS on the genotypes' antioxidative abilities by measuring guaiacol peroxidase (GPX), and catalase (CAT) enzyme activities. GPX activity significantly differed between genotypes, whereas CAT enzyme activity was comparable between the genotypes under drought stress treatment (Fig. 2B and Table S1C). Increased rate of guaiacol-peroxidase (GPX) activity has been previously correlated with the drought tolerance of plants [38]. The increased GPX response in *MM11* indicates its ability to mitigate oxidative stress during reproductive stage drought stress.

The yield, physiological and biochemical attributes under drought stress showed a significant positive correlation of NFG, NSP, PH, proline, WUEi and Pn with grain yield per plant (GYP) (Fig. S1). The correlation of all the above growth attributes of *MM11* during reproductive stage drought stress suggests that increased plant height, panicle length, number of filled grains per panicle, number of spikelets per panicle (NSP), proline content, better water use efficiency and photosynthesis rate, together facilitate *MM11* mutant plants to sustain the grain yield during drought stress condition.

### Transcriptome-based analysis of yield and drought-related DEGs in *MM11* panicle

**Fig. 3:**
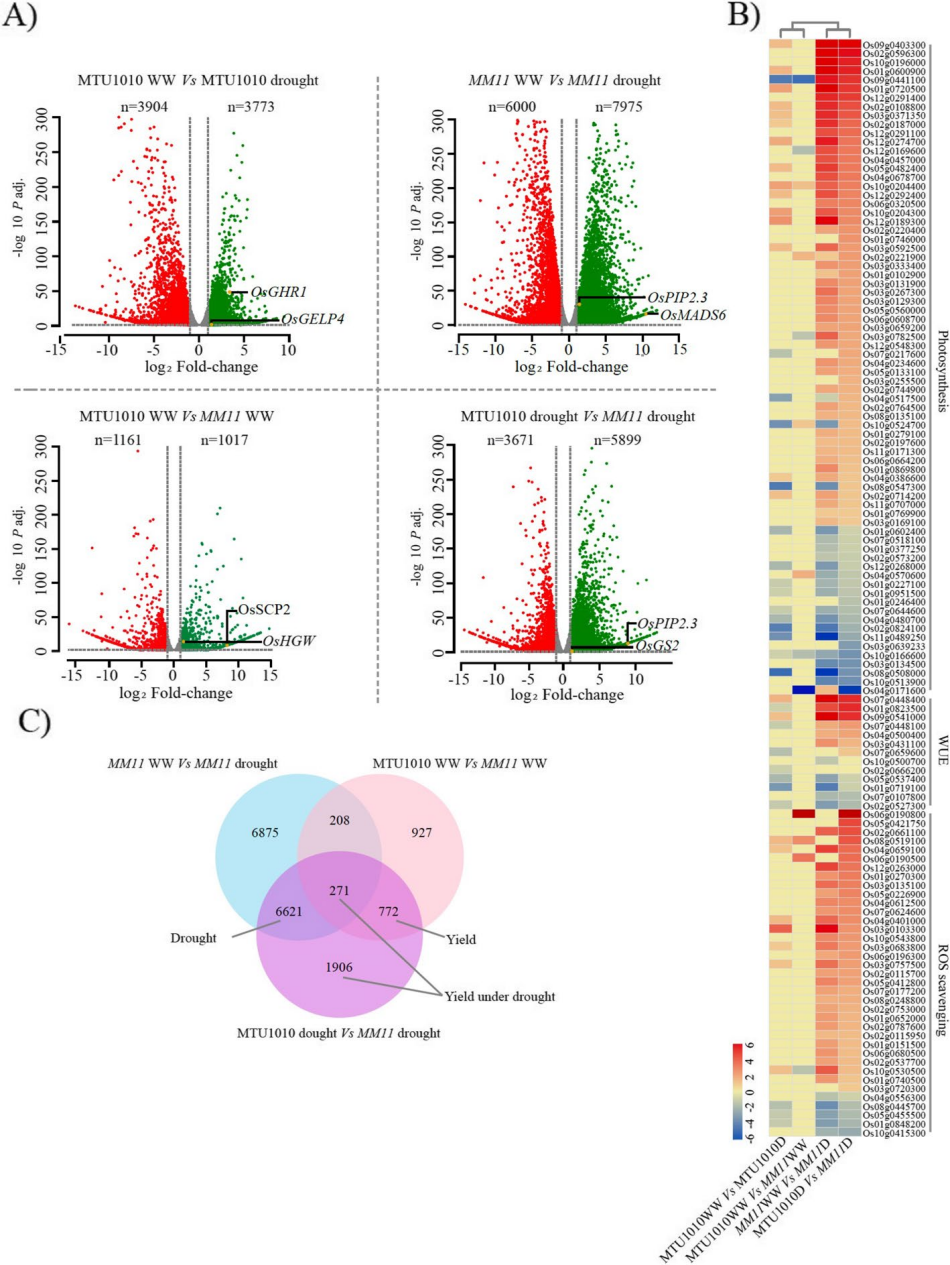
RNA-seq data analysis from panicle tissues of MTU1010 and *MM11* subjected to reproductive stage drought stress. In response to drought stress, significant (fold change > 2, *p* < 0.05) differentially expressed genes (DEGs) were categorized into yield, drought and yield under drought genes based on common and unique gene expression. **A** Volcano plot representing DEGs across the WT and mutant in well-watered (WW) and drought conditions; x-axis shows fold-change (log_2_) difference in the expression, and y-axis shows the negative log of adjusted *p*-value for the expression. Non-significant genes are indicated by grey dots. Red and green color indicates downregulated and upregulated DEGs. ‘n’ indicates total number of significant DEGs. The yellow dot indicates known genes for yield and drought from which few of them were further validated with qPCR expression analysis. **B** The expression of genes involved in drought-responsive biological processes was compared in MTU1010 and *MM11* under well- WW (MTU1010 WW/*MM11* WW) and drought (MTU1010 D/*MM11* D) conditions. **C** Significant DEGs were categorized into yield, drought and yield under drought genes based on the overlapping and unique gene expression in mutant under drought as well as well-watered (WW) conditions with respect to MTU1010

Next, we did a comparative transcriptomic analysis of *MM11* and MTU1010 emerged panicles under well-watered (WW) and drought stress conditions to understand the molecular players underlying the physiological changes in *MM11* plants during the reproductive stage drought stress response. The RNA-seq analysis resulted in approximately 1.26 billion quality-filtered reads, with an average of 1.05 billion reads per biological sample. Filtered reads from 12 libraries had a mapping rate of 91.3% to 92.5% when mapped against the rice genome (Table S2A). Unbiased principal component analysis (PCA) was executed on all RNA sequencing samples to determine the distinct gene profiles. It was observed that, the replicates were clustered differently in each condition and showed a strong positive correlation among the samples (Fig. S2). Using a FDR of < 0.05 and log_2_FC cut-off criteria of > 1 and < -1, we identified significantly up-regulated genes and down-regulated genes respectively in four different comparisons (i) MTU1010 WW *vs* MTU1010 drought (7,677 DEGs), (ii) *MM11* WW *vs MM11* drought (13,975 DEGs), (iii) MTU1010 WW vs *MM11* WW (2,178 DEGs) and (iv) MTU1010 drought *vs MM11* drought (9,570 DEGs) (Fig. 3A and Table S2B). Consistent with the morphological and physiological changes (Fig. 2B), several drought-responsive genes involved in biological processes such as photosynthesis, WUE and ROS scavenging are strongly regulated in *MM11* under drought conditions as compared to MTU1010 (Fig. 3B). We categorised the DEGs into yield (Y), drought (D) and yield under drought (YD) genes (Fig. 3C). The yield (Y) category consists of genes that are commonly differentially expressed in the *MM11* panicle compared to MTU1010 in the well-watered condition (MTU1010 WW *vs MM11* WW) and drought condition (MTU1010 drought *vs MM11* drought). DEGs common in both (i) drought imposed *MM11* panicle compared to those grown in control conditions (*MM11* WW *vs MM11* drought) and (ii) drought stress imposed *MM11* panicle versus drought imposed MTU1010 panicle (MTU1010 drought *vs MM11* drought) were categorized as drought (D) category. Finally, the yield under the drought (YD) category encompassed the (i) unique genes which are only differentially expressed in a drought-treated *MM11* panicle to drought treated MTU1010 panicle, and (ii) DEGs that were common among in drought-imposed or WW *MM11* and MTU1010 and DEGs in *MM11* well-watered *vs* drought (Fig. 3B). Such categorisation led to the identification of 772 yield genes, 6621 drought genes, and 2177 yield under drought genes. We then annotated the DEGs using KEGG and MAPMAN ontology to map metabolic and regulatory changes, respectively among the three categories of DEGs (Fig. S3). KEGG mapping revealed a metabolism overview with the distribution of DEGs mainly in carbohydrate, lipid, terpenoid, amino acid, nucleotide and energy metabolism, whereas MAPMAN ontology distributed DEGs in regulation categories such as transcription factor, protein modification and degradation, hormones and signalling. The extent of overall metabolic changes in represented categories followed D > YD > Y pattern (Fig. S3), which was proportional to the number of DEGs in each category, suggesting lower intrinsic yield responsive genes and an expected large number of drought-responsive DEGs between parent and mutant panicles.

**Fig. 4:**
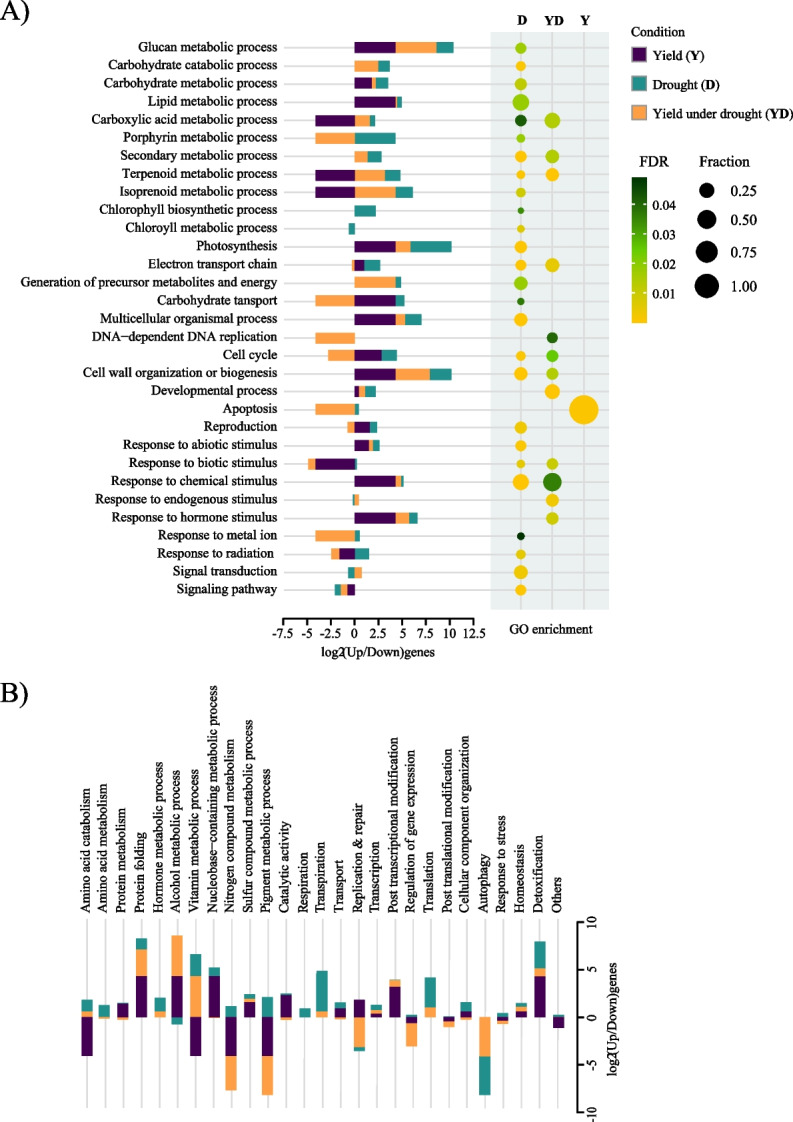
Gene ontology enrichment analysis of yield, drought, and yield under drought DEGs. The GO annotation analysis of DEGs from drought (D), yield under drought (YD) and yield (Y) categories, classified them into different biological processes (BP). **A** The x-axis represents biological processes either categorized (log2up/down) or enriched (FDR < 0.05). The y-axis represents ratio of upregulated and downregulated DEGs categorized in BP or significance for enrichment in BP. The enrichment of BP is indicated by color scale of yellow to green (high to less significant) and by different circle sizes depending on the number of genes enriched. **B** The log2 ratio of upregulated and downregulated D, YD and Y DEGs in BP those were not significantly enriched. The color distinction for D, YD and Y category is shown at the upper right corner

GO annotations and enrichment of the three categories of DEGs were performed to obtain an overview of biological processes with functional relevance in each category (Fig. 4A, Tables S2C and S2D). Based on the number of genes in each category, the expected number of biological processes (BP) enriched was found to be more in D (39 BP), than in YD (17 BP), and in Y (4 BP), which are again broadly classified into D (26), YD (12) and Y (1). For instance, DEGs from the drought category were significantly enriched in photosynthesis, response to abiotic stress, intracellular signalling cascade, multicellular organismal process, reproduction, phenylpropanoid metabolic and polysaccharide catabolic process. On the other hand, yield under drought DEGs was enriched in response to hormone stimulus and developmental processes. A few common BP terms were enriched in D and YD categories, such as electron transport chain, cell wall organization, terpenoid metabolism process, and cell cycle. Unexpectedly, a large proportion of DEGs in the yield category were significantly enriched in apoptosis. Even though a considerable subset of DEGs were found to be significantly enriched in GO biological processes, however quite a few DEGs with assigned GO terms were not part of any of the enriched GO terms (Fig. 4B). GO enrichment analysis revealed several drought-responsive biological processes and apoptosis as the most significant processes to affect the yield in *MM11*. Also, it indicated that developmental processes and hormone stimulus could play an essential role in sustaining yield under drought stress.

### Regulatory candidate genes for yield and drought stress

**Fig. 5:**
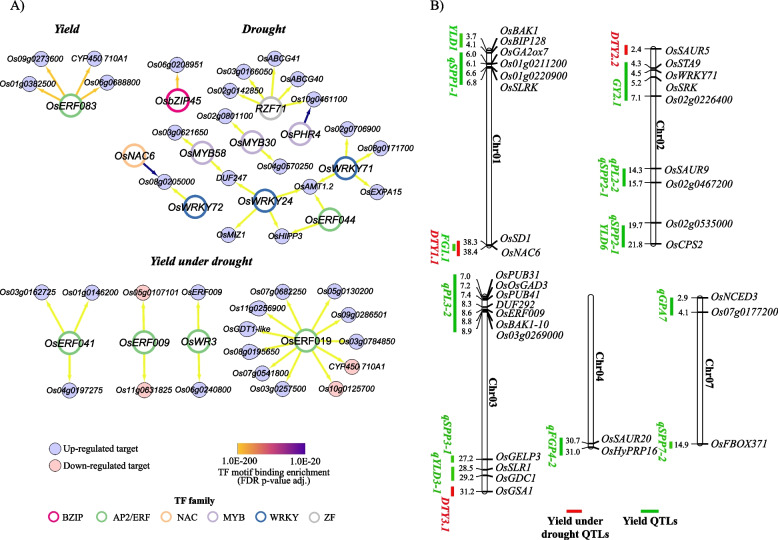
Inferred gene regulatory connections and co-localization of candidate DEGs in known yield and yield under drought QTLs. **A** Regulatory interactions based on drought induction in high yield mutant panicle predicted for yield, drought and yield under drought related DEGs. Target genes are indicated in smaller nodes where blue and red represents upregulated and downregulated DEGs, respectively. Larger source node represents transcription factors that significantly regulate target genes based on expression pattern and their binding site enrichment in target gene promoters. Border color of source node represents TF family classification. Edge connections are colored in the shades of yellow to purple according to the magnitude of binding site enrichment significance (FDR *p*-value adj.). **B** Chromosomal positions of several DEGs from RNA seq analysis along with few DEGs from TF-target interaction, co-localized with known QTLs for yield and drought. Green line indicates yield related QTLs and red indicate drought QTLs. Blue colored genes are DEGs from the interaction networks. On each chromosome, the gene corresponding values are the physical distances in Mb

Reconstructing gene regulatory networks is crucial in understanding complex biological processes such as yield. After analysing the DEGs, we aimed to identify the regulatory components that could be involved in yield enhancement and sustained yield under drought in the *MM11* panicle. To this end, we analysed highly significant TF-target interactions in yield (Y), drought (D) and yield under drought (YD) DEGs using a regression tree algorithm RTP-STAR [39]. In total, the initial inferred network obtained 90 TFs with 1888 targets for drought DEGs, 24 TFs with 273 targets for yield under drought DEGs and 10 TFs with 251 targets for yield DEGs (Table S3A). Further, enrichment of TF motif binding sites in the 1 kb promoters of each DEG from three categories was performed to refine the final network. Only the TFs and their targets overlapped between TF motif binding site enrichment analysis and inferred network from RTP-STAR analysis were retained (Table S3B). This resulted in a massive reduction of TF and targets in each category, resulting in highly significant regulatory interactions. The final drought network contained 10 TFs with 17 targets that involved TFs belonging to MYB (*OsMYB58*, *OsMYB30*, and *OsPHR4*), AP2/ERF (*OsERF044*), NAC (*OsNAC6*), BZIP (*OsbZIP45*), ZF (*OsZF71*) and WRKY (*OsWRKY24*, *OsWRKY71*, and *OsWRKY72*) TF family. Out of all the targets, only seven were annotated (*OsAMT1.2*, *OsHIPP3*, *OsMIZ1*, *OsEXPA15*, *DUF247*, *OsABCG40*, and *OsABCG41*), and the other 10 were unannotated DEGs (Fig. 5A). The yield under the drought network contained four TFs belonging to the AP2/ERF TF family (*OsERF041*, *OsERF009*, *OsWR3*, and *OsERF019*) that were predicted to target three annotated DEGs (*OsGDT1-like*, *CYP450710A*, and *OsERF009*) and 15 unannotated DEGs. Lastly, the yield network contained only one TF belonging to the AP2/ERF TF family (*OsERF083*) with one annotated target (*CYP450710A*) and three unannotated targets. Among all the three TF-target networks, *OsERF019* had the highest number of targets (11 DEGs), followed by *OsERF083* and *OsZF7*, and with four targets each. The TF-target interactions provide a few novel TFs and targets that could pinpoint biological significance in yield and drought signalling.

We next mapped, all the DEGs onto the rice genome to analyse their co-localisation with known yield and drought QTLs (Fig. 5B). A total of 33 candidate DEGs were found to be co-localised in 17 QTLs on chromosomes 1, 2, 3, 4 and 7 (Fig. 5B). Among them, 15 yield QTLs were identified, such as qSPP1-1, qYLD1, FG1.1, GY2.1, PL2.2, qSPP2-1, YLD6, qFGP4-2, qSSD4-1, qPL3-2, qSPP3-1, qYLD1-1, qGPA7, qSSP7-1, and qSSP7-2. For yield under the drought category, DTY1.1, DTY2.2 and DTY3.1 QTLs were identified (Table S4B). The majority of the DEGs were co-localised in yield QTLs, whereas genes such as *OsSD1*, *OsNAC6*, *OsGSA1* and *OsSAUR5* were found to overlap within the yield under drought QTLs. Interestingly, two TFs (*OsWRKY71* and *OsERF009*) from the TF-target regulatory networks were also co-localised within the yield QTLs. The presence of many significant DEGs and predicted TF-target gene modules among the co-localised genes in well-known yield/drought-related QTL regions show the robustness and possible mechanistic underpinnings of the genetically determined QTLs of the study (Figs. 3 and 5A).

**Fig. 6:**
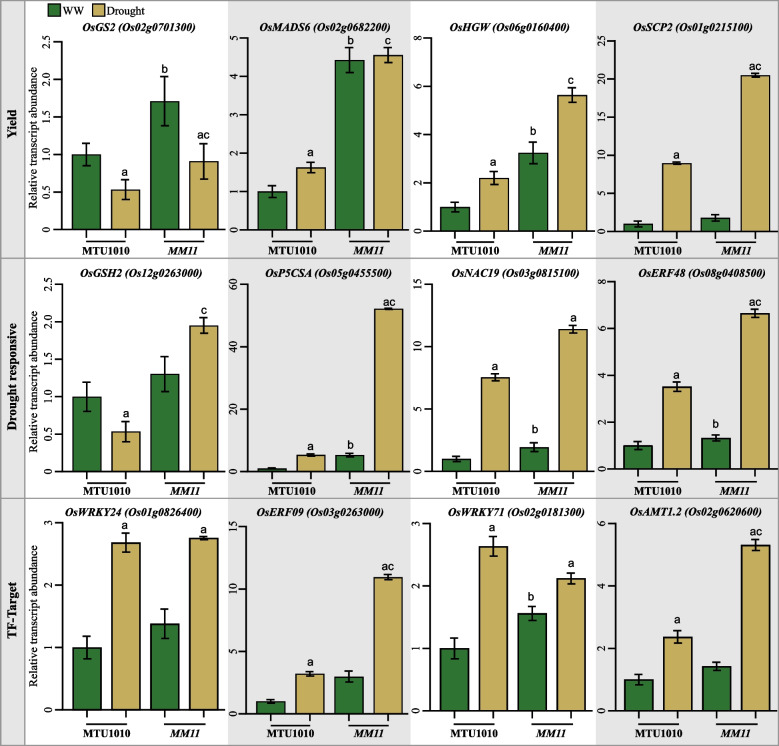
Quantitative real-time PCR validation of yield and drought DEGs. Expression analysis of few previously studied, yield related genes, drought responsive genes and TF-target candidate genes in MTU1010 and *MM11* under well-watered (WW) and drought conditions indicated similar differential expression pattern compared to RNA seq analysis. Each plot shows relative transcript abundance of known marker genes across MTU1010 and *MM11* under WW and drought conditions. Each bar represents the mean of six biological replicates along with indicated standard error of the mean. ‘a’ indicates significant variation (*p* < 0.05) in transcript abundances between MTU1010 WW and MTU1010 drought or *MM11* WW and *MM11* drought; ‘b’ indicates variation between MTU1010 WW and *MM11* WW; ‘c’ indicates variation between MTU1010 drought and *MM11* drought. The statistical details are given in supplementary Table S4A

For validation of RNAseq-derived DEGs, some previously reported genes involved in improving rice yield and drought tolerance mechanisms (Figs. 3A and B) and predicted TF-target interactions from this study (Fig. 5A) were chosen. In Table S4A, the expression profile of these genes in the various RNAseq comparison sets is represented as a table. Using RT-qPCR, the expression profile of these 18 selected genes was validated in the young panicles of MTU1010 and *MM11* grown under control and drought conditions (Fig. 6 and Table S4A and C). Several genes reported to impart increased yield and drought tolerance were differentially regulated in *MM11* under well-watered (WW) and drought conditions compared to MTU1010. For example, RNAseq analysis identified a rice *plasma membrane intrinsic protein2;3* (*OsPIP2;3*) as differentially expressed in *MM11* under control and drought conditions (Fig. 3A). Consistent with this finding, RT-qPCR analysis revealed that *OsPIP2;3* is fivefold upregulated in MTU1010 in response to drought treatment. In contrast to MTU1010, the basal level of *OsPIP2;3* expression in *MM11* is already threefold higher under control conditions and significantly increases under drought conditions (Table S4A). Similarly, Similarly, the basal level expression of rice *abscisic acid, stress and ripening5* (*OsASR5*) is also increased in *MM11* compared to MTU1010 under control conditions (Table S4A). When it comes to yield-related genes, the transcript abundance of *GS2* (*OsGRF4*), which controls grain shape, panicle length [40] and *HGW*, maintaining heading date and grain weight [41] and *MADS6*, a positive regulator of the lodicule-, stamen- and carpel-like organs in rice [42], were increased significantly in *MM11* compared to MTU1010 under drought conditions (Fig. 6). In addition, a serine carboxypeptidase 2 (SCP2), which is a homologue of *Grainsize5* (*GS5/OsSCP26*), a major QTL determining rice grain size [43], and *SCP46*, a positive regulator of grain filling in rice [44] was also significantly upregulated in *MM11* under drought compared to MTU1010 (Fig. 6). These findings explain the better adaptability of *MM11* reflected in yield-related traits such as PL, NFG and NSP in well-watered (WW) and drought conditions compared to MTU1010 (Figs. 2 and 6).

Abiotic stress causes the accumulation of metabolites such as glutathione and proline to protect the plant from the adverse effects of stress. Increased levels of these metabolites are positively correlated with increased tolerance to drought [37, 38]. The glutathione and proline biosynthesis genes, *glutathione synthetase* (*GSH2*) and *Δ1-pyrrolin-5-carboxylate synthetase* (*P5CSA*), respectively, were significantly upregulated in *MM11* than WT under drought conditions (Fig. 6). In rice, members of the TF families, such as AP2/ERF, MYB, NAC, and WRKY, play an important role in rice drought stress response [45, 46]. Our RNAseq analysis identified two TFs (NAC domain family, *OsNAC19* and ERF family, *OsERF48*) to be differentially regulated in *MM11* than its parent MTU1010. Furthermore, our TF-target interaction networks also identify multiple TFs belonging to NAC and ERF TF family and their targets (Fig. 5). The expression profile of a NAC domain family TF *OsNAC19* and ERF family TF *OsERF48* are significantly upregulated in *MM11* in comparison to MTU1010 (Fig. 6). Overexpression of TFs has been implicated in the root system architecture and thereby conferring increased drought tolerance in rice [14]. Further, we tested our predicted TF-target gene module by validating the expression of *OsAMT1.2*, which encodes ammonium transporter-1.2, and is a common target of *OsWRKY71* and *OsWRKY24*. In addition, we also validate another TF OsERF09 that might contribute to yield under drought (Fig. 5). The transcript of *OsAMT1.2* was more than twofold increase in MM11 under drought compared to MTU1010. Similarly, the transcript level of *OsERF09* showed ~ threefold increase in *MM11* in comparison to MTU1010 under drought conditions (Fig. 6). *OsERF09* is directly regulated by *OsNAC10* and contributes the drought tolerance in rice [47]. Collectively transcript analysis of selected target genes shows that *MM11* is able to withstand drought and contributes to increased yield by increased expression of the genes responsible for drought tolerance. Overall, the physiological and molecular characterization of *MM11* during the reproductive stage drought stress, revealed a few novel regulatory candidates that could be important in sustaining higher yield during rice panicle development.

## Discussion

Reproductive stage drought stress (RDS) is detrimental to overall rice yield [29, 48, 49]. It is thus essential to understand the molecular mechanisms that could govern yield under drought stress and provide drought resistance at the reproductive stage. In the present study we adopted a mutational breeding approach to generate a gamma-irradiated population of the MTU1010 rice variety. We identified five high-yielding mutant genotypes in subsequent generations. These high-yielding genotypes show an increased grain yield (up to 50.87%) mainly due to the increase in the number of filled grains and a higher number of spikelets per panicle in comparison to MTU1010 (Fig. 1). Among the high-yielding genotypes, we selected the *MM11* genotype for evaluation of its response to reproductive stage drought stress because it consistently exhibited superior yield-related traits across generations. (Fig. 1 and Table S1). Consistent with its field-level performance, *MM11* displayed increased yield performance under the pot culture method (47.38%) compared to MTU1010 under well-watered (WW) conditions. Further, when subjected to prolonged drought stress at the panicle emergence stage (reproductive), *MM11* sustained a high yield advantage of 38.29% compared to MTU1010. We quantified several morphological, photosynthesis-related and biochemical changes associated with reproductive drought stress in *MM11* and compared it to MTU1010 (Fig. 2B). Results indicate that the increased yield under drought in *MM11* is probably due to enhanced photosynthesis ability, high osmoprotection and better instantaneous water use efficiency compared to MTU1010. We found several photosynthesis-related GO terms such as photosynthesis, chlorophyll biosynthesis, and chlorophyll metabolic process were enriched in *MM11* compared to MTU1010 (Table S2C). Different mechanisms such as ROS accumulation, and diminished CO_2_ influx exist through which the rate of photosynthesis under drought stress is suppressed [50]. Here, the mutant line *MM11* showed better photosynthetic rate and WUE as well as higher expression levels of photosynthetic responsive genes (Fig. 3B). A strong positive selection on photosynthetic genes conferred rice with better drought tolerance, with fewer declines in photosynthesis rate [51]. Overall, panicle development actively integrated with drought-induced changes at the reproductive stage such as reduced transpiration rate and reinforced its role in increasing the sink capacity and water use efficiency to fulfil considerable grain filling [52].

At the molecular level, drought stress has been studied in various rice tissues, majorly in leaves. Several genes implicated in drought stress tolerance have been well characterized [53]. In general, reproductive tissues such as panicles exhibit better flexibility under water shortages than leaves at the physiological and molecular level, mainly due to their stability of photosynthetic apparatus, better primary metabolism and water status [54]. However, prolonged drought stress during the reproductive stage is detrimental to rice yield. Very few studies have been reported on the molecular players involved in reproductive tissues, such as panicles during drought stress [25]. In this study, we performed a comparative panicle transcriptome analysis between MTU1010 and *MM11* to identify the genes and gene regulatory networks underlying the better and sustained yield performance of *MM11* mutant under RDS. The DEGs in response to RDS have been classified under three categories, Yield contributing genes (Y), Drought response genes (D), and genes that are responsible for sustained yield under drought (YD) (Fig. 3). When we look into the drought-responsive genes, they all typically belonged to biological processes related to photosynthesis, ROS scavenging and WUE (Fig. 3B). This result is consistent with the growth trait measurements that validate the robustness of the drought response. For instance, anticipated ROS production upon drought stress, resulted in increased proline accumulation and expression of genes related to the antioxidant system, including (GO:0009628) *Os01g0270300*, *Os06g0196300* and *Os02g0537700* (GO:0098869) in *MM11* that could negatively regulate the growth inhibition responses [55]. Interestingly, these findings mirror those for in wheat and barley reproductive tissues [56].

Further comparison of DEGs in MTU1010 and *MM11* panicle transcriptomes under yield (Y), drought (D), and yield under drought (YD) categories revealed distinct biological processes operating under these three categories. GO analysis revealed significant enrichment of shared and distinct biological processes (BP) between the three categories (Fig. 4). Upon RDS, drought (D)-specific response showed significant enrichment of a large number of the BP in panicle compared to Y and YD categories. These processes are related to metabolism (Glucan, carbohydrate and lipid), energy, signal transduction and reproduction. Carbohydrate assimilation processes represent one of the natural responses to regulate energy balance in plants under drought stress [57]. Among them, genes involved in trehalose biosynthesis and transport (*Os02g0753000*, *Os07g0624600*, *Os02g0661100* and *Os07g0485000*) were differentially expressed in *MM11* compared to MTU1010 (Table S2C), suggesting this BP could be vital in the protection and adaptation of panicle against drought-induced stress.

In the YD category, we found significant and specific enrichment of BP related to the developmental process and hormone stimulus was found specifically, suggesting their role in improving yield under drought stress in *MM11*. Most of the DEGs in developmental processes were involved in regulating vegetative to reproductive phase transition and cell differentiation, indicating improved panicle development in *MM11* during RDS. Whereas, under hormone stimulus BP, DEGs were majorly related to auxin, ethylene and ABA-activated signalling pathways (Table S2C). During the early panicle development in rice, auxin plays an essential role in forming transition meristems [58]. In contrast, high ABA and low ethylene have been suggested as adaptive traits for spikelet fertility upon drought stress [59]. In addition, co-expression of genes related to auxin, ethylene and ABA has been observed during drought response in sugarcane leaves [57]. Consistent with this observation, overexpression of TFs integrating ethylene and ABA signalling has conferred improved drought tolerance and yield in soybean [60]. Likewise, it is plausible that similar hormone signalling components identified in the emerged panicle of *MM11* contribute to enhanced grain filling during RDS.

Besides, few BP were significantly enriched in both D and YD categories such as carboxylic acid metabolism, secondary metabolism, electron transport chain, cell cycle, cell wall biogenesis and response to a chemical stimulus. This highlights the importance of panicles in producing organic and amino acids for developing grains. Accumulation of amino acids in panicles is known to protect the cells from photo-inhibition under drought stress [61, 62]. Thus, metabolic activity and cellular growth could undergo reprogramming under drought stress in panicles to integrate drought-responsive genes and yield betterment [63]. DEGs in the Y category were unrelated to drought response since they contained genes uniquely expressed in *MM11* panicles under control conditions. GO enrichment analysis showed a significant association of Y category DEGs only in BP related to cell death, apoptosis, belonging to NBS-LRR or ARC domain-containing proteins. NBS-LRR/ARC-related proteins are generally known for their role in biotic disease resistance by participating in the hypersensitive response, a form of localized programmed cell death [64]. Their role in emerged panicle development has yet to be determined. However, it has been shown before that RDS dramatically affects male sterility by ABA and GA-mediated programmed cell death (PCD) in anthers [29, 30]. The NBS-LRR/ARC-related proteins identified in our study might be necessary for programmed cell death during male flower development and an increased spikelet fertility in *MM11* under RDS. Based on the findings presented here, we envision that studying the roles of these genes in spikelet fertility, panicle development and grain filling will pave the way to new regulatory/signalling mechanisms involved in yield maintenance.

Among the genetic modifications, TF-based regulations have received significant attention for their role in abiotic stress tolerance [65]. Regulatory aspects of drought stress responses in *MM11* were assessed by identification of significant TF-target interactions (Fig. 5B). The analysis highlighted the role of a few novel and known TF families (NAC, WRKY, MYB and AP2/ERF) that are active during drought stress responses [66]. For instance, our analysis identified a drought-specific regulatory network, wherein the binding site of *OsWRKY71* was found to be significantly enriched in promoters of DRGs such as *OsAMT1.2* (encodes membrane protein, ammonium transporter-1.2), *OsEXPA15* (encodes cell-wall protein, expansin-A15), *Os08g0171700* and *Os02g0706900*. This predicted TF (*OsWRKY71*)-target gene (*OsAMT1.2*) has been validated in our study by RT-qPCR (Fig. 6). *OsAMT1.2* and *OsEXPA15* are involved in BP, such as nitrogen metabolism and cell wall organization, and consistently these BPs are enriched in yield under the drought responses (YD) category. Previously, WRKY TFs, such as *OsWRKY78* and *SiLP1* has been characterized for their role in panicle development and grain yield [67, 68]. Thus, *OsWRKY71* which is also located in grain yield-related QTL region (GY2.1) could be an important candidate target gene for improving ammonium allocation and cell expansion during emerged panicle development under drought stress. Besides, other TFs such as *OsERF009* and *OsNAC6* were found in yield-related and yield under drought-related QTL regions, respectively. Both AP2/ERF (*OsERF3* and *OsERF4*) and NAC TF (*OsNAC5* and *OsNAC6*) family members have been characterized for their role in yield and drought responses [19, 69–71], however their role in panicle is not known. *OsERF009* was found to putatively regulate two unannotated genes (*Os11g0631825* and *Os05g0107101*), whereas, *OsNAC6* binding site was significantly enriched in the promoter of *Os08g0205000* that encodes an uncharacterized transferase domain-containing protein. These uncharacterized genes could be important for studying yield under drought responses in rice panicle. Importantly, our regulatory network outlays high confidence TF-target interactions that could be involved in integrated signalling between emerged panicle development, yield and drought stress.

## Conclusion

In conclusion, we envisage that the *MM11* mutant, generated in this study maintained a higher yield under RDS can be used as a donor in rice breeding programs targeted to achieve sustained yield under reproductive stage drought stress. Furthermore, once genome information of *MM11* will be available then it can be considered for a new variety release program for rainfed and direct seeded conditions after rigorous field testing and multilocation field trials. In addition, we perform a comparative emerged panicle transcriptome to unravel the molecular players contributing to yield and yield under drought in *MM11*. Our study identifies several key regulatory genes governing yield and drought stress and their interactions. We construct gene regulatory networks (GRNs) based on DEGs in *MM11* and identify potential TF-target gene modules that might contribute to yield and drought, and validate some TF-candidate gene modules. Several candidate genes identified in our study are colocalized to known QTLs governing yield and drought. These can be excellent choices for functional characterization and studying their contribution to the respective QTL.

## Materials and methods

### Mutant development and selection

Seeds of MTU1010 (500 g) were procured from Andhra Pradesh Rice Research Institute (APRRI), Maruteru, India. Gamma rays were applied at a dose of 250 Gy in the Gamma Cell 220 irradiator at Bhabha Atomic Research Centre, Mumbai, India. The M1 plants were raised from mutagenized (M0) seeds of MTU1010 in the wetland farm of S.V. Agricultural College at geographical coordinates of 13°54’ N latitude and 79°54’ E longitude, and 182.9 m altitude in Tirupati, Acharya N. G. Agricultural University (ANGRAU), India, during Kharif-2015 (June to November) along with wild-type MTU1010. These M1 plants were protected from outcrossing and harvested individually to obtain the M2 seeds. At each subsequent generation from M2 onwards, significantly stable mutant lines were advanced for the subsequent generation cultivation. A total of 280 families were selected for propagation into the M3 generation from winter (Rabi) -December 2016-April-2017. In the M3 generation, eight mutant lines were selected based on plant type and grain character similar to wild-type and propagated into the M4 generation and screened for high yielding attributes during Monsoon (Kharif; June to November), 2018. The experiment was laid in a randomized complete block design (RCBD) with three replications, with the spacing of 20 cm between rows and 15 cm between plants within the row per line. Non-segregating five mutant lines that showed relatively stable yield traits during M4 and M5 generations (Rabi, 2018–19) were selected and further advanced to the subsequent generation cultivation.

### Field evaluations for selected high-yield mutant lines

The selected high-yield M5 mutant lines designated as *MM* (*MTU1010 derived Mutant*) included *MM11*, *MM73*, *MM151*, *MM152*, and *MM155*. These lines were propagated to the M6 generation during Kharif-2019. At the maturity stage, various yield-related traits were recorded. Such as days to maturity (DTM); the days were calculated from sowing to maturity, Plant height (PH, in cm); length of the plant from the ground surface to the tip of the tallest panicle, Panicle length (PL, in cm); distance between panicle neck node bases to the last spikelet’s tip, Number of spikelets per panicle (NSP); the number of spikelets were counted for each panicle, Number of filled grain number per panicle (NFG); Seed setting rate or spikelet fertility (SF); the ratio of filled grain number per panicle to spikelets per panicle, 1000-grain weight (TGW); after drying in the air, randomly five samples of 1000 grains were taken from filled grains, weighed and recorded and total grain yield per plant (GYP) were recorded or calculated, as appropriate. Considering the above parameters from the five mutant lines, we selected the mutant line *MM11* owing to its better yield attributes across seasons and subsequently evaluated its performance under reproductive stage drought stress condition in pot culture in rainout shelter during Rabi (December to May), 2019–20.

### Reproductive stage drought stress treatment

During Rabi-2018–19, MTU1010 and *MM11* lines were subjected to the reproductive stage drought stress under field condition for 21 days and evaluated yield and yield related traits were evaluated under field conditions. The plot size of each genotype was 1.0 m × 0.5 m, with five rows with a spacing of 20 cm between rows and 15 cm between plants within the row per genotype, and the two treatments, i.e., well-watered (control) and stress plots. The two treatment plots were separated by 2.0 m space. The water seepage from the control plot to the stress plot was restricted by making a huge channel lined up with a plastic sheet (2 m width) in the 2.0 m space. Soil moisture tension was recorded using Tensiometer (the model 2710ARL). A tensiometer was installed in the soil at a depth of 30 cm, and soil moisture tension was recorded on a daily basis. As per standard protocol, when the pressure reaches 15 psi, life-saving irrigation was given in the stress plot. In addition we also performed, a pot experiment in a complete randomized design with two treatments (well-watered (WW) and drought-stressed), two lines (MTU1010 and mutant line, *MM11*) under a rainout shelter during Rabi, 2019–20. All these experiments were conducted in three biological replicates. Ten pre-germinated seeds of the two lines were initially sown on round pots (24 cm length × 22.5 cm width × 21.5 cm height) filled with 15 kg of a mixture of farmyard manure (20%) and soil (80%). Upon seedling establishment, three healthy seedlings were retained in a pot and grown until the booting stage. A day before imposing stress, all the pots were saturated with water and allowed to drain excess water so that the soil moisture in each pot would be uniform. The moisture stress was imposed by withholding water at the booting stage (reproductive stage) in the stress pots for 14 days. The moisture percentage was measured in each pot using the moisture probe (Lutron PMS-714 model) at 25 cm depth. After 14 days of moisture stress, when the moisture content reached 10–15%, the stressed pots were re-watered daily until the plants attained physiological maturity. Regular irrigation was given to the control plants throughout the crop growth period. On the 14^th^ day of moisture stress, the morphological, physiological, and biochemical traits were recorded in control and stress-imposed pots. Plants were harvested at the stage of physiological maturity, and yield-related traits were quantified.

### Quantification of physiological and biochemical parameters

The leaf water relations were estimated in the form of relative water content (RWC) as described previously [72]. Briefly, the fully expanded fresh leaf samples of 500 mg of each MTU1010 and mutant lines from control and drought stress plants were used to estimate the RWC. Fresh weight (FW) was obtained immediately after the leaves were excised from the plant in the morning. Then, they were kept in 10 ml of distilled water in a falcon tube at room temperature for about 12 h to obtain turgid weight (TW). Subsequently, each leaf sample was put in the oven (model NKOA-3, India) for 48 h at 80^◦^C to attain the dry weight (DW). The values of the fresh weight (FW), turgid weight (TW), and dry weight (DW) were used to calculate the relative water content (RWC).

$$RWC\left(\%\right)= \left[\frac{(FW\left(g\right)-DW\left(g\right))}{(TW\left(g\right)-DW\left(g\right))}\right]x 100$$

Specific leaf area (SLA) was recorded for five flag leaves collected from all positions of a canopy of five plants grown under both control and drought stress conditions. The leaf area (LA) was estimated using a leaf area meter (model LI-3100; LI-COR, Lincoln, NE, USA). The leaves were dried at 80 °C in a hot air oven, and the leaf dry weight (LDW) was taken [73, 74].

$$SLA\left({cm}^{2}{g}^{-1}\right)= \frac{(LA)}{(LDW)}$$

Gas exchange measurements such as photosynthetic rate (Pn), transpiration rate (E), stomatal conductance (gs), and intercellular CO_2_ concentrations (Ci) were measured after 14 days of moisture stress in both control and stressed plants (Six plants of each genotype per treatment) using the LI-6400 gas exchange portable infrared CO_2_ analyzer (IRGA; ADC, Bio scientific Ltd, Hoddesdon, UK). With Pn, E, gs, and Ci values, instantaneous water use efficiency (Pn/E = WUEi) and the intrinsic WUE (Pn/gs = iWUE) were calculated.

Proline content was investigated using L-proline as standard as described by Chen and Zhang [75]. Briefly, crude protein extracted from 200 mg of fresh flag leaves was mixed with 1 mL reaction solution containing 10 mL 3% sulphosalicylic, 10 mL acetic acid, and 20 mL 2.5% acidicninhydrin. The mixed solutions were boiled at 100 °C for 15 min. After cooling to room temperature, absorbance at 520 nm was measured and the proline level of samples was calculated by making the specification curve with the known concentration of L-proline. The malondialdehyde (MDA) content was measured as described previously by Chen and Zhang [75]. 200 mg of frozen flag leaf for each sample was ground in 3 mL of 100 mM PBS (pH 7.8) and centrifuged for 10 min at 12,000 rpm. In a 1.5 mL centrifuge tube, 100 µL of supernatant from each sample was combined with 1 mL of 0.25 percent TBA solution. The reaction mixture was incubated at 100 °C in a water bath for 15 min with 1 mL 0.25 percent TBA solution and 100 mL of 100 mM PBS (pH 7.8) as a reference. After cooling down the reaction mixture on ice, the absorbance of the supernatant at 532 and 600 nm was determined with a spectrophotometer (Biotek Synergy H1 hybrid multimode reader). MDA content was calculated by the extinction coefficient (155 mmol^−1^ cm^−1^) of MDA-TBA at 532 nM.

For the antioxidant enzyme assay, a pre-chilled mortar and pestle were used to grind approximately 200 mg of fresh flag leaf tissues, and the leaf powder was homogenized by adding three mL of 100 mM PBS buffer (pH 7.8). The homogenate was centrifuged for 10 min at 4 °C at 10,000 g. The supernatant was retrieved as a crude enzyme extract and assayed for antioxidative enzyme activity. Catalase (CAT, EC 1.11.1.6) and guaiacol peroxidase (GPX, EC 1.11.1.7) activities were assayed as described previously by Chen and Zhang [75].

### RNA extraction and sequencing

For RNA isolation, three biological replicates of emerged panicle tissue samples were snap-frozen and collected from the drought stress pots and normal irrigated pots after 14 days of stress period at the reproductive stage (heading stage at ~ 15% moisture percentage). The samplings were performed in the morning, around 10–11:30 am. RNA was extracted from panicle tissue of both MTU1010 and *MM11* for well-watered and drought stress conditions using RNAIso Plus (Takara Bio Inc) reagent following the manufacturer’s instructions. RNA was treated with a the TURBO DNA-*free* ™ kit (Life Technologies), according to the manufacturer’s protocol. RNA quality was determined by 1% RNA agarose gel and further by using Agilent 2100 Bioanalyzer (Santa Clara, CA, USA). The samples that passed the quality check were used for library preparation. Sequencing libraries were prepared using an Illumina TruSeq® RNA Library Preparation Kit (Illumina®, San Diego, CA, USA) as per the manufacturer’s protocol and run on an Illumina NovaSeq6000.

### RNAseq analysis

In total, 12 samples of cDNA libraries were prepared for paired-end sequencing (including three biological replicates) for each treatment of two lines; MTU1010 and *MM11*. After RNA sequencing raw reads were generated and converted into FASTQ format. These FASTQ file quality checks were done by using the FastQC tool (http://www.bioinformatics.babraham.ac.uk/projects/fastqc/). Reads with Phred score > 20 were considered for further analysis. Pre-processing (removal of adapter and low-quality reads) of files was done by Cutadapt [76] and the trim galore tool (http://www.bioinformatics.babraham.ac.uk/projects/trim_galore/). Again, the quality check was done by FastQC. Sequence alignment was carried out by the Hisat2 tool [77] with the reference genome (Oryza_sativa.IRGSP-1.0.dna.toplevel.fa) obtained from Ensembl Plants (http://plants.ensembl.org//). Conversion of SAM to BAM format was done using the SAM tool (version 1.11) as well as sorting and indexing. Using HTseq (https://github.com/simon-anders/htseq), we generated the count files. The expression level of the genes was estimated by using fragments per kilobase of transcript per million mapped reads (FPKM). Differentially expressed genes (DEGs) were identified by using the R package DEseq2. DEGs were filtered out by using a threshold of the fold change of more than 2 (log_2_FC of 1) and FDR value < 0.05. FDR value (adjusted *P*-value) was calculated by using the Benjamin-Hochberg method of correction for multiple testing. Gene ontology term for each DEG was taken from GO slim in Ensembl plant Biomart. These GO terms were manually classified into broader terms to plot graphs for Biological processes, molecular functions and cellular components. Gene Ontology (GO) enrichment analysis was performed using AgriGO V2 to identify enriched GO term. The DEGs were mapped onto metabolic pathways using the KEGG pathway analysis tool to identify which cellar pathways are enriched in different conditions and genotypes [78]. Pathway analysis was done by using the MapMan visualization tool (WWW.https://mapman.gabipd.org/mapman.). MapMan analysis was focused on the regulatory pathway that broadly covers major categories of biological regulation.

### Prediction of TF-target interactions

For TF-target network inference, a random forest approach (RTP-STAR) was used to analyse the replicate data within the Tuxnet interface ([39]; https://github.com/rspurney/TuxNet). Distinct networks were obtained for yield (Y), drought (D) and yield under drought (YD) categories using respective DEGs and expression values in different biological samples and their replicates. Rice-specific TF data file was downloaded from http://planttfdb.gao-lab.org/index.php?sp=Osj and provided for the run so as to identify the TFs present in DEGs. With ten iterations, an edge proportion of 0.33, and average expression values, TF-target edges were generated. All the three networks were combined by taking the union of RTP-STAR output files. The TF and their targets in RTP-STAR network output files were further filtered on the basis of TF motif binding site enrichment analysis. For enrichment analysis, 1 kb upstream sequences from the transcription start site of each DEG were mined from the genome of rice (RAP-DB). The promoter sequences were then scanned for the enrichment of TF binding sites using the MEME suite AME web tool by mapping on rice-specific TF meme file (downloaded from http://planttfdb.gao-lab.org/index.php?sp=Osj) with default parameters except, *e*-value report threshold was changed to 2000. Three MEME output files were generated for each category (Y, D, and YD). These outputs were merged and only significant enrichments were included (*p*-value adj. < 0.001). Further, both RTP-STAR output and MEME output were analysed and only those TF and their targets were retained that overlapped between both the outputs. The retained output was used to generate the final network file to be visualized in Cytoscape.

### The reverse transcription quantitative real-time PCR (RT-qPCR)

RNA (1 μg) was treated with DNaseI and reverse-transcribed using an iScriptTM selected cDNA synthesis kit (Biorad), and the resulting cDNA was used for RT-qPCR analysis. The RT-qPCR analysis, which was carried out in a 10 μl reaction mix with 2µL of template cDNA, 5µL of qPCR SYBR Green Master Mix (Biorad), 0.5µL (5 pmol) of forward and reverse gene-specific primers, each and 2µL of Milli-Q water. Rice *18S* rRNA and *actin* genes were used as the internal reference control and gene-specific primers were used for the relative expression of selected genes. All the primers were listed in Table S4C.

### Statistical analysis

In all figures, the data are expressed as mean standard error mean (SEM). Student *t*-test, and one-way analysis of variance (ANOVA) were performed using IBM SPSS 20.0v software to determine the significance of difference for all traits, and the charts were drawn by Graphpad Prism 9 software. Pearson’s Correlation analysis between traits was computed at *p* ≤ 0.05 and *p* ≤ 0.01 using OriginPro (Origin Lab Corporation, Northampton, MA, USA).

### Supplementary Information

**Additional file 1: Supplementary Figure S1.** Correlation between Yield, physiological and biochemical growth attributes. **Supplementary Figure S2.** Qualitative analysis of RNA seq data from MTU1010 and *MM11* (mutant) panicle under drought stress. **Supplementary Figure S3.** Analysis of D, YD and Y category DEGs for mapping into metabolic pathways and regulatory components.

**Additional file 2: Table S1A.** Field evaluations of MTU1010 and 5 mutants (*MM11, MM151, MM152, MM155 *and *MM73*) for Yield attributes. **Table S1B.** Field evaluations of MTU1010 and *MM11* under drought stress during winter 2018-19.** TableS1C.** Pot culture evaluations of MTU1010 and *MM11* under drought stress. **Table S2A.** Summary of filtered and mapped reads for RNA seq sample. **Table S2B.** DEGs in response to Drought stress in MTU1010 (WT) and *MM11 *(Mutant). **Table S2C.** GO term annotation and categorization of biological processes for DEGs. **Table S2D.** GO enrichment of drought (D), yield under drought (YD) and yield (Y) DEGs. **Table S3A.** Tuxnet RTP-STAR output for Yield, drought and yield under drought DEGs.** Table S3B.** Overlapping TF-target interactions between tuxnet RTP-STAR output and MEME-AME analysis. **Table S4A.** Statistical calculations for RT-qPCR data assesment. **Table S4B.** Chromosomal locations and identifiers of each QTL analysed. **Table S4C.** List of primer sequences used for RT-qPCR analysis. The F and R represents forward and reverse primer sequences of each gene.

## Data Availability

All data supporting the findings of this study are available within the paper and its supplementary data. RNA-seq data was deposited on the Sequence Read Archive (https://www.ncbi.nlm.nih.gov/sra/) under BioProject accession number PRJNA865102.

## Abbreviations

RDS: Reproductive stage drought stress
YD: Yield under drought
Pn: Photosynthetic rate
WUEi: Water use efficiency
NSP: Number of spikelets per panicle
NFG: Number of filled grain per panicle
MDA: Malondialdehyde
CAT: Catalase
DEGs: Differentially expressed genes
BP: Biological processes
TF: Transcription factor
