# Supplementary material for: Panicle transcriptome of high-yield mutant indica rice reveals physiological mechanisms and novel candidate regulatory genes for yield under reproductive stage drought stress
Source: BMC Plant Biol. 2023 Oct 13;23:493. doi: 10.1186/s12870-023-04507-1 (PMC10571340; doi:10.1186/s12870-023-04507-1)
Supplement: Supplementary file 1 — Additional file 1: Supplementary Figure S1. Correlation between Yield, physiological and biochemical growth attributes. Supplementary Figure S2. Qualitative analysis of RNA seq data from MTU1010 and MM11 (mutant) panicle under drought stress. Supplementary Figure S3. Analysis of D, YD and Y category DEGs for mapping into metabolic pathways and regulatory components. [file 12870_2023_4507_MOESM1_ESM.pdf]

Additional file 1:

Supplementary Figure S1. Correlation between Yield, physiological and biochemical growth attributes. Pearson coefficients comparison among yield attributes (NSP, number of spikelet/panicle; NFG, number of filled grain/plant; PH, plant height; PL, panicle length; SF, spike fertility; TSW, thousand seed weight; GYP, grain yield/plant; NT, number of tillers and CH, culm height), physiological (Pn, photosynthesis rate; Ci, intercellular CO_2 level; RWC, relative water content; gs, stomatal conductance; WUEi, water use efficiency and SLA, specific leaf area) and biochemical attributes (Proline; MDA, malondialdehyde; CAT, catalase and GPX, guaiacol-peroxidase) in MTU1010 and MM11 under well-watered (WW) and drought condition. Significant values are indicated on the right side of each plot with positive and negative correlations in red and blue color, respectively.

Supplementary Figure S2. Qualitative analysis of RNA seq data from MTU10101 and *MM11* (mutant) panicle under drought stress. Principal component analysis (PCA) was performed for four comparisons between MTU1010 and mutant under well-watered (WW) and drought conditions. X and Y-axes represent variation among the replicates. Blue and red dots indicate WW and drought stressed samples, respectively. Each dot in the PCA plot is denoted by the sample names, for example, WW_1S, where S indicates sample.

Supplementary Figure S3. Analysis of D, YD and Y category DEGs for mapping into metabolic pathways and regulatory components. KEGG mapping and MAPMAN ontology categorized DEGs into metabolism overview and regulation overview components. In the metabolism overview (left panel), complete metabolic pathways are green colored and mapped pathways are in red. Different colored boxes indicate type of metabolism. In regulation overview (right panel), MAPMAN output represents categorization of DEGs as small bins in regulatory components such transcription factor, hormones, light etc. Intensities of blue and red color in bins represent upregulation and downregulation of mapped DEGs respectively**.**

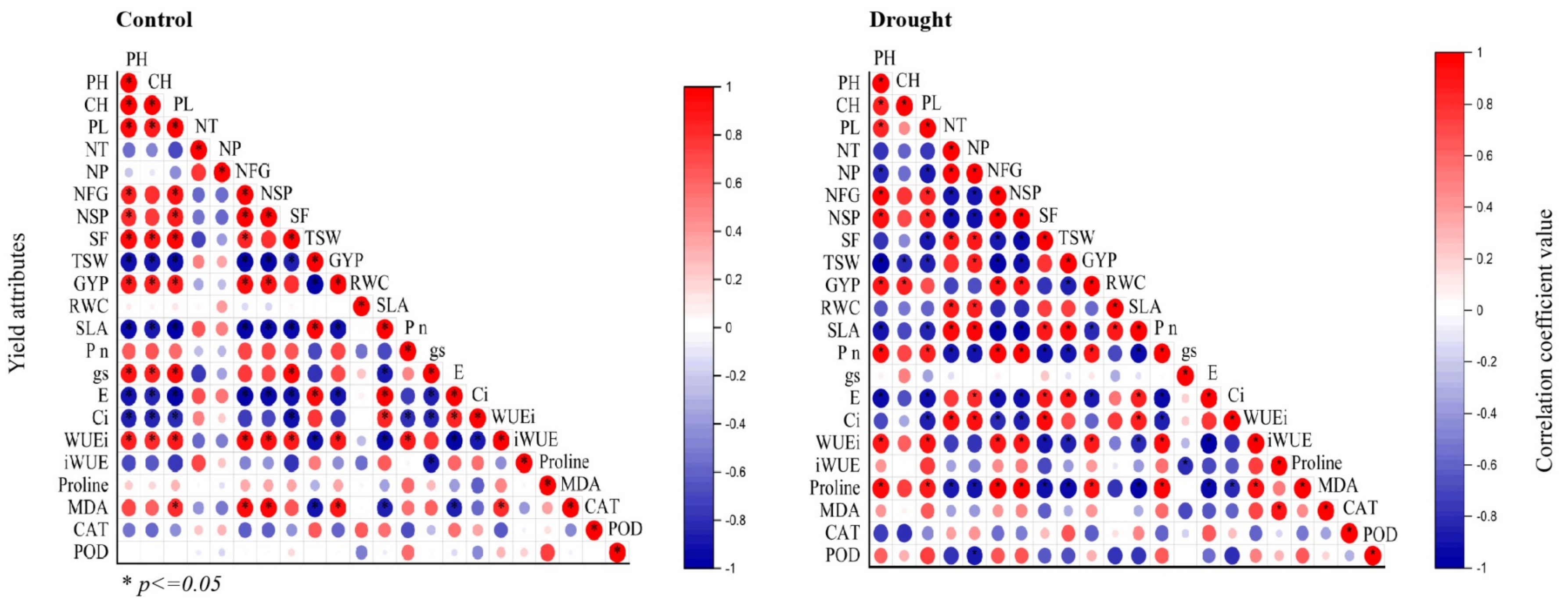

Supplementary Figure S1. Correlation between Yield, physiological and biochemical growth attributes. Pearson coefficients comparison among yield attributes (NSP, number of spikelet/panicle; NFG, number of filled grain/plant; PH, plant height; PL, panicle length; SF, spike fertility; TSW, thousand seed weight; GYP, grain yield/plant; NT, number of tillers and CH, culm height), physiological (Pn, photosynthesis rate; Ci, intercellular CO_2 level; RWC, relative water content; gs, stomatal conductance; WUEi, water use efficiency and SLA, specific leaf area) and biochemical attributes (Proline; MDA, malondialdehyde; CAT, catalase and GPX, guaiacol-peroxidase) in MTU1010 and MM11 under well-watered (WW) and drought condition. Significant values are indicated on the right side of each plot with positive and negative correlations in red and blue color, respectively.

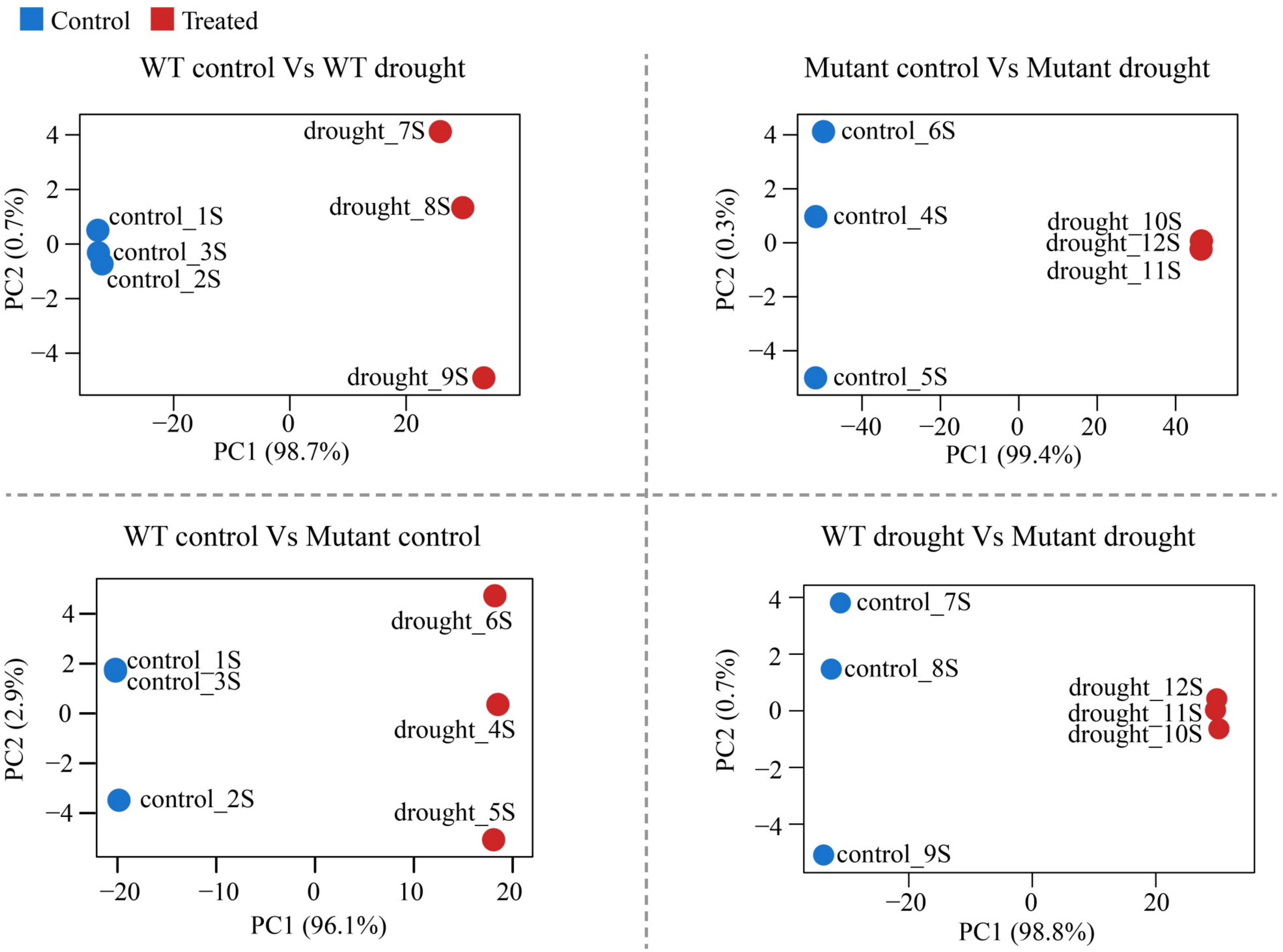

Supplementary Figure S2. Qualitative analysis of RNA seq data from MTU10101 and *MM11* (mutant) panicle under drought stress. Principal component analysis (PCA) was performed for four comparisons between MTU1010 and mutant under well-watered (WW) and drought conditions. X and Y-axes represent variation among the replicates. Blue and red dots indicate WW and drought stressed samples, respectively. Each dot in the PCA plot is denoted by the sample names, for example, WW_1S, where S indicates sample.

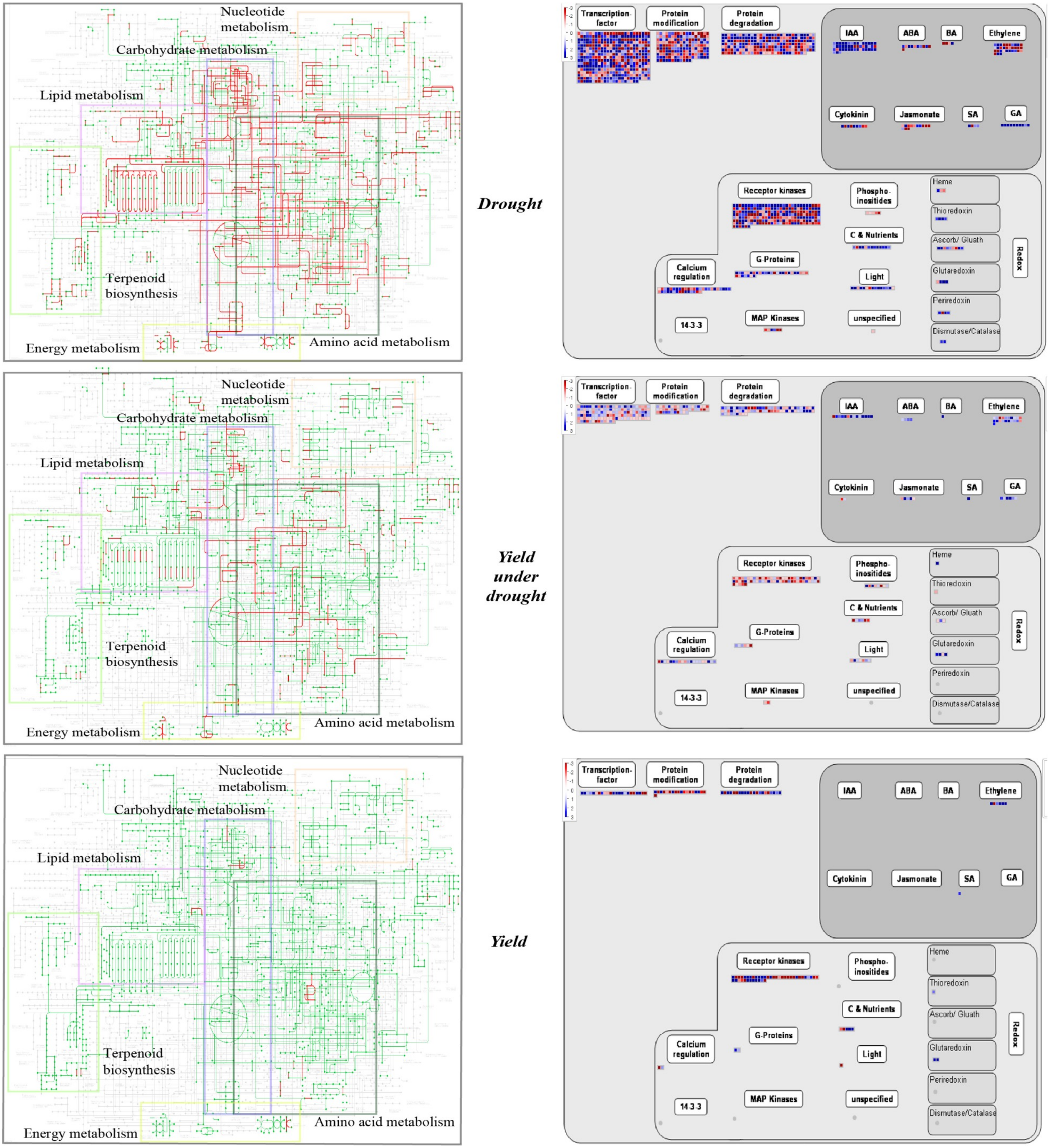

Supplementary Figure S3. Analysis of D, YD and Y category DEGs for mapping into metabolic pathways and regulatory components. KEGG mapping and MAPMAN ontology categorized DEGs into metabolism overview and regulation overview components. In the metabolism overview (left panel), complete metabolic pathways are green colored and mapped pathways are in red. Different colored boxes indicate type of metabolism. In regulation overview (right panel), MAPMAN output represents categorization of DEGs as small bins in regulatory components such transcription factor, hormones, light etc. Intensities of blue and red color in bins represent upregulation and downregulation of mapped DEGs respectively.
